# Effect of prepaid and promised financial incentive on follow-up survey response in cigarette smokers: a randomized controlled trial

**DOI:** 10.1186/s12874-019-0786-9

**Published:** 2019-07-04

**Authors:** Yee Tak Derek Cheung, Xue Weng, Man Ping Wang, Sai Yin Ho, Antonio Cho Shing Kwong, Vienna Wai Yin Lai, Tai Hing Lam

**Affiliations:** 1 School of Nursing, The University of Hong Kong, Hong Kong, China; 2 School of Public Health, The University of Hong Kong, Hong Kong, China; 3 The Hong Kong Council on Smoking and Health, Hong Kong, China

**Keywords:** Incentive, Follow-up, Randomized controlled trial, Smoker

## Abstract

**Background:**

Monetary incentive is often used to increase response rate in smokers’ survey, but such effect of prepaid and promised incentives in a follow-up survey is unknown. We compared the effect of different incentive schemes on the consent and retention rates in a follow-up survey of adult cigarette smokers.

**Methods:**

This was a randomized controlled trial (RCT) in Hong Kong, China. Smokers who completed a non-incentivized baseline telephone smoking survey were invited to a 3-month follow-up, with randomization into (1) the control group (no incentive), (2) a promised HK$100 (US$12.8) incentive upon completion, (3) a promised HK$200 (US$25.6) incentive upon completion, or (4) a prepaid HK$100 incentive plus another promised HK$100 incentive (“mixed incentive”). Crude risk ratios from log-binomial regression models were used to assess if the 3 incentive schemes predicted higher rates of consent at baseline or retention at 3-month than no incentive.

**Results:**

In total, 1246 smokers were enrolled. The overall consent and retention rates were 37.1 and 23.0%, respectively. Both rates generally increased with the incentive amount and offer of prepaid incentive. The mixed incentive scheme marginally increased the retention rate versus no incentive (26.8% vs 20.3%; risk ratio (RR) = 1.32; 95% CI: 1.00–1.76; *P* = 0.053), but not the consent rate (RR = 1.13; 95% CI: 0.93–1.38; *P* = 0.22). Among the consented participants, approximately 50% in the mixed incentive group received the mailed prepaid incentive, who achieved a higher retention rate than the group without incentives (82.8% vs 56.1%; RR = 1.48; 95% CI: 1.21–1.80; *P* < 0.01).

**Conclusion:**

The mixed incentive scheme combining the prepaid and promised incentive was effective to increase the follow-up retention rate by 48%. We recommend this mixed incentive scheme to increase the follow-up retention rate. More efficient methods of delivering the incentive are needed to maximize its effects.

**Trial registration:**

U.S. Clinical Trials registry (clinicaltrials.gov, retrospectively registered, reference number: NCT03297866).

## Background

Tobacco control polices require representative and longitudinal cohort samples for rigorous evaluation. Although telephone surveys remain an effective approach for population-based surveillance, conducting a representative telephone survey has become more difficult in recent years owing to household landlines being increasingly replaced by mobile phones, the wide use of answering devices and voicemail, and frequent telemarketing in an over-surveyed society like Hong Kong [1–5]. Recent observational studies face increasing challenges from nonresponses [6–8], which may introduce nonresponse bias [9, 10] in estimating public opinions for policy-making.

Loss to follow-up in tobacco control cohort surveys is an ongoing challenge. Non-respondents tend to have lower socioeconomic status [11] and have no intention to quit smoking [12]. Hence, monetary incentives are often promised for completing follow-up surveys, and larger incentives have yielded higher response rates [13, 14]. However, large incentives may not be practical due to budget constraints.

Prepaid incentives appear to be a feasible alternative to promised incentives for mail [15, 16], face-to-face [17], and telephone surveys [18]. The Social Exchange Theory posits that offering prepaid incentive fosters a trusting relationship with the participants, who would feel obliged to reciprocate by responding to the follow-up survey [19, 20]. In addition, the behavioral theory on present-biased preferences suggests that current potential rewards and costs have a larger impact than those in the future [21]. Therefore, incentive schemes with prepaid and earlier rewards should be more attractive than other schemes with the same amount but delayed rewards [22, 23]. To the best of our knowledge, no study has rigorously tested the effect of prepaid incentive on smokers’ follow-up retention, and little is known about the effect of payment amount or timing on follow-up survey retention.

This was the first randomized controlled trial (RCT) aimed to assess the effect of payment amount (HK$100 (US$12.8) or HK$200 (US$25.6)) and payment timing (prepaid or promised) on smokers’ consent and completion of a follow-up survey at 3 months. We hypothesized that (1) the effects of incentive increase with the amount offered and that (2) a combination of prepaid and promised incentive yields better response than no incentive.

## Methods

### Trial design and subjects

This was a parallel 4-armed, individual RCT with equal allocation ratio, which was nested within the Tobacco Control Policy-related Survey (TCPS) 2017. The TCPS 2017 is a representative, cross-sectional, telephone opinion survey (baseline survey) on tobacco control in Hong Kong [24], where smoking prevalence has declined from 23.3% in 1982 to 10.0% in 2017 [25]. Approximately 25% of smokers in Hong Kong are hardcore smokers [26, 27]. The baseline survey adopted a two-stage random sampling strategy by initially randomly drawing landline telephone numbers from residential telephone directories and then selecting the adult (aged 18 years or above) family member who had the next birthday soonest. The survey was administered by a trained interview survey agent. Details of the survey have been reported elsewhere [28, 29]. Respondents who self-reported current use of cigarettes were eligible for the RCT. Before each survey, they gave verbal informed consent with the knowledge that the survey data were for quantitative analysis and that the interview would be audio-recorded. The baseline survey of the RCT was conducted from late June to mid-October 2017. All follow-up interviews were completed by late January 2018. This study has been approved by the Institutional Review Board of the University of Hong Kong/Hospital Authority Hong Kong West Cluster (IRB reference number: UW 17–084) and registered in the U.S. Clinical Trials registry (reference number: NCT03297866).

### Survey incentives

We set the basic incentive to HK$100 (US$12.8), which was equivalent to 3 h of minimum wage in 2017 (HK$34.5 (US$4.4)) and was greater than those in previous trials of prepaid incentive targeting the general population [30–32].

After completing the baseline survey, all eligible participants were invited for a similar 3-month follow-up survey with a question: “The School of Nursing and School of Public Health of the University of Hong Kong would like to contact you later to collect more opinion on tobacco health warnings and other tobacco control policies. Would you like to participate? If you agree, please tell me your contact number and the research staff will contact you soon.” They were randomly allocated using the survey system run by the survey agency (Public Opinion Programme of the University of Hong Kong) into one of the 4 groups of incentive schemes: (1) no incentive, (2) a promised HK$100 incentive (i.e., a HK$100 supermarket voucher after completing the follow-up survey), (3) a promised HK$200 incentive (i.e., a HK$200 supermarket voucher after completing the follow-up survey), and (4) a mixed incentive (i.e., a HK$100 supermarket voucher for agreeing to participate in the follow-up survey and another HK$100 supermarket voucher after completing the follow-up survey). Respondents allocated to receive incentives (groups 2–4) were informed of the respective incentives to encourage their participation.

All consented participants were asked to provide only the telephone number for re-contact but not also the postal address because we considered that collecting too many personal details may deter their consent to the follow-up. Participants in the mixed incentive group who consented to the follow-up were re-contacted twice at most within 2 weeks to collect the mailing address for the pre-survey incentive. Those who could not be reached were deemed to have not successfully received the incentive, but they were still contacted for the follow-up survey and were given HK$200 incentive for completing it. All follow-up telephone interviews were conducted by experienced interviewers blinded to the group allocation. Each respondent was called up to 7 times at different time slots within 1 month. All incentives were sent using registered mails.

### Randomization, allocation concealment and blinding

Randomization was conducted using a computerized interview system of the survey agent after the respondents confirmed their smoking status and gave informed consent, hence the group allocation was concealed to both interviewers and participants before randomization. Both interviewers and the participants were not blinded about their respective intervention (i.e. incentive scheme), but the 3-month follow-up interviewers were blinded.

### Outcomes

The primary outcomes were (1) the consent rate (i.e., the proportion of participants who consented to the 3-month follow-up at baseline) and the (2) retention rate (i.e., the proportion of participants who completed the 3-month follow-up). Adopting the intention-to-treat principle, both outcomes were assessed using all eligible and consented participants as the denominator. To distinguish the effect between receiving and not receiving the prepaid incentive, we added the retention rate among the consented participants as an additional outcome, which was not stated in the original protocol.

### Statistical analyses

The sample size was constrained by the limited baseline survey period and limited funding; thus, the optimal sample size (*n* = 1336 for a risk ratio of 1.33 comparing 2 groups, power = 80%, and alpha = 0.05) in examining the effectiveness of prepaid and promised incentives was not achieved. We used Chi-square test to assess if the socio-demographic characteristics were balanced among the trial groups. We reported risk ratios from log-binomial regression model instead of odds ratios from logistic regression, because the outcome events in this study were common (proportion of 20% or more). Crude risk ratios from log-binomial regression models were used to assess if the three incentive schemes yielded higher rates of consent or retention than no incentive. Predictors with *P*-values below 0.1 in univariate analysis were included in multivariate log-binomial regression models to generate adjusted risk ratios. Interactions between the incentive schemes and other significant predictors were then included in the models. All data analyses were conducted using IBM SPSS Statistics, version 23. A two-sided *P*-value less than 0.05 was considered statistically significant.

## Results

A total of 1246 participants who reported currently smoking were invited at baseline, and were included in the final analysis. The number of subjects in the 4 trial groups were 316, 326, 283 and 321. Of all participants, 84% were men and the participant mean age was 55.1 years. Most participants attained secondary education level, were either married or cohabitating, employed, had children, were daily cigarette smokers, and born in Hong Kong (Table 1). Approximately 25% smoked their first cigarette within 5 min after waking, and approximately 50% reported no intention to quit smoking. No significant difference in baseline sociodemographic and smoking characteristics was noted among the 4 arms (all *P*-values > 0.05).

**Table 1:** Sample characteristics according to incentive schemes at baseline (*n* = 1246)

	No incentive (*n* = 316)	Promised HK$100 incentive (*n* = 326)	Promised HK$200 incentive (*n* = 283)	Mixed incentive (*n* = 321)	*P*-value
Sex					0.82
Male	264 (83.8)	277 (85.0)	243 (85.9)	268 (83.5)	
Female	51 (16.2)	49 (15.0)	40 (14.1)	53 (16.5)	
Age group (years)					0.49
< =39	59 (18.9)	51 (15.6)	51 (18.0)	64 (19.9)	
40–59	132 (42.0)	134 (41.1)	102 (36.0)	129 (40.2)	
> =60	123 (39.2)	141 (43.3)	130 (45.9)	128 (39.9)	
Education level					
Primary or below	63 (20.2)	71 (21.8)	69 (24.4)	69 (21.6)	0.80
Secondary	189 (60.6)	188 (57.7)	159 (56.2)	178 (55.8)	
Tertiary	60 (19.2)	67 (20.6)	55 (19.4)	72 (22.6)	
Marital status					0.57
Single	63 (20.2)	53 (16.3)	50 (17.9)	66 (20.8)	
Married / Cohabit	222 (71.2)	233 (71.5)	198 (70.7)	215 (67.8)	
Divorced/Separated/Widowed	27 (8.7)	40 (12.3)	32 (11.4)	36 (11.4)	
Employment status					0.31
Employed	182 (58.1)	180 (55.4)	139 (50.4)	186 (58.3)	
Retired	100 (31.9)	119 (36.6)	105 (38.0)	100 (31.4)	
Others	31 (9.9)	26 (8.0)	32 (11.6)	33 (10.3)	
Monthly household income (HKD; US$1 = HK$7.8)					0.29
Below $10,000	65 (23.4)	57 (19.8)	54 (21.9)	52 (18.7)	
$10,000–$29,999	95 (34.2)	99 (34.4)	101 (40.9)	96 (34.5)	
$30,000 or above	118 (42.4)	132 (45.8)	92 (37.2)	130 (46.8)	
Had children	219 (71.3)	246 (76.4)	209 (75.5)	220 (69.8)	0.19
Had children under 16 years	46 (15.0)	65 (20.3)	55 (20.0)	54 (17.1)	0.28
Place of origin					0.57
Hong Kong	181 (58.4)	188 (58.6)	164 (58.8)	200 (63.5)	
China Mainland	118 (38.1)	127 (39.6)	108 (38.7)	105 (33.3)	
Others	11 (3.5)	6 (1.9)	7 (2.5)	10 (3.2)	
Perceived health status					1.00
Extremely /very good	85 (27.6)	99 (30.8)	79 (28.4)	92 (29.1)	
Good	83 (26.9)	80 (24.9)	79 (28.4)	82 (25.9)	
Fair	115 (37.3)	117 (36.4)	97 (34.9)	118 (37.3)	
Bad	25 (8.1)	25 (7.8)	23 (8.3)	24 (7.6)	
Time to first smoking after waking					0.57
<5 min	72 (26.2)	76 (25.5)	71 (28.1)	75 (25.8)	
6–30 min	69 (25.1)	84 (28.2)	71 (28.1)	91 (31.3)	
31–60 min	37 (13.5)	29 (9.7)	31 (12.3)	40 (13.7)	
After 60 min	97 (35.3)	109 (36.6)	80 (31.6)	85 (29.2)	
Daily cigarette users					0.11
Yes	256 (82.1)	250 (77.2)	240 (84.8)	261 (81.6)	
No	56 (17.9)	74 (22.8)	43 (15.2)	59 (18.4)	
Daily cigarette consumption (sticks)					0.17
1–10	149 (52.5)	150 (53.8)	111 (43.4)	145 (49.8)	
11–20	114 (40.1)	115 (41.2)	126 (49.2)	118 (40.5)	
21–30	13 (4.6)	11 (3.9)	13 (5.1)	16 (5.5)	
>30	8 (2.8)	3 (1.1)	6 (2.3)	12 (4.1)	
Intention to quit					0.93
No intention	169 (54.9)	172 (54.6)	153 (55.4)	177 (56.5)	
Within 30 days	49 (15.9)	54 (17.1)	41 (14.9)	42 (13.4)	
After 30 days	90 (29.2)	89 (28.3)	82 (29.7)	94 (30.0)	
Ever used Ecigs or HTP					0.24
Yes	72 (22.8)	72 (22.1)	50 (17.7)	78 (24.3)	
No	244 (77.2)	254 (77.9)	233 (82.3)	243 (75.7)	

Mixed incentive: Prepaid HK$100 incentive and promised HK$100 incentive; Ecigs or HTP: E-cigaretes or heated tobacco products; Sample sizes varied because missing responses on some variables were not included in the percentage calculation. *P*-value calculated by Chi-squared test for categorical variables. All differences were due to chance (from randomization), and *P* values are for reference only

**Table 2:** Consent and retention rates at 3-month follow-up according to incentive schemes (*n* = 1246)

Incentive schemes	Consent rate *n* (%)	Risk ratio (95% CI)	*P*-value	Retention rate *n* (%)	Risk ratio (95% CI)	*P*-value
All (*n* = 1246)	462 (37.1)			287 (23.0)		
No incentive	114/316 (36.1)	Ref		64/316 (20.3)	Ref	
Promised HK$100	110/326 (33.7)	0.94 (0.76, 1.16)	0.54	69/326 (21.2)	1.05 (0.77, 1.41)	0.78
Promised HK$200	107/283 (37.8)	1.05 (0.85, 1.29)	0.66	68/283 (24.0)	1.19 (0.88, 1.60)	0.27
Mixed incentive	131/321 (40.8)	1.13 (0.93, 1.38)	0.22	86/321 (26.8)	1.32 (1.00, 1.76)	0.053
Participants who consented (*n* = 462)				287 (62.1)		
No incentive	–	–	–	64/114 (56.1)	Ref	
Promised HK$100	–	–	–	69/110 (62.7)	1.12 (0.90, 1.39)	0.21
Promised HK$200	–	–	–	68/107 (63.6)	1.13 (0.91, 1.41)	0.20
Mixed incentive not receiving prepaid	–	–	–	33/67 (49.3)	0.88 (0.66, 1.18)	0.38
Mixed incentive receiving prepaid	–	–	–	53/64 (82.8)	1.48 (1.21, 1.80)	<0.01

HK$100 = US$12.8; Incentive-mix: Prepaid HK$100 incentive and promised HK$100 incentive

Overall, 462 (37.1%) participants consented to the follow-up and provided their mobile or landline telephone number for further contact (Table 2). Of them, 287 (23.0%) completed the follow-up survey. The mixed incentive group had the highest consent rate (40.8%), followed by the promised $200 incentive group (37.8%), although they were not significantly different from the no incentive or control group (36.1%). The retention rates increased with the amount of incentives (no incentive, 20.3%; $100 incentive, 21.2%; $200 incentive, 24.0%) and was highest for the mixed incentive group (26.8%). Compared with the control group, the mixed incentive group showed a higher retention rate (RR = 1.32, 95% CI: 1.00–1.76; *P* = 0.053), with marginal significance.

Of the 131 participants who consented to the follow-up in the mixed incentive group, 64 (48.9%) received the prepaid incentive. The other 67 participants did not receive the prepaid incentive, including 50 (74.6%) who could not be contacted by the subsequent follow-up interviewer to provide a mailing address, 14 (20.9%) who refused to provide their mailing address but agreed to the later follow-up, and 3 (3.4%) who refused the later follow-up. The retention rate was higher in those who received the prepaid incentive (82.8%) than in those who did not (49.3%).

Among the participants who consented to the follow-up, the retention rate was significantly higher in those who received the prepaid incentive in the mixed incentive group (82.8%) than that in the control group (56.1%) (adjusted RR (ARR) = 1.48, 95% CI: 1.21–1.80; *P* < 0.01). Meanwhile, the participants in the mixed incentive group who did not receive the prepaid incentive had a lower retention rate than the control group (49.3% vs 56.1%); however, the ARR was non-significant (ARR = 0.88, 95% CI: 0.66–1.18; *P* = 0.38) (Table 2).

In the multivariate models, higher education level, being born in Hong Kong (versus Mainland China and other places), and having an intention to quit were associated with both higher consent and retention rates (Table 3). Female sex (versus male sex) and having children aged under 16 years were associated only with higher consent rate. All interaction terms were not significant.

**Table 3:** Predictors of consent at baseline and completion of the 3-month follow-up (*n* = 1246)

	Consented to follow-up		Completed follow-up	
Predictors	Adjusted risk ratio (95% CI)	*P*-value	Adjusted risk ratio (95% CI)	*P*-value
Incentive schemes		0.18		0.20
No incentive	Ref		Ref	
Promised HK$100	0.92 (0.75–1.13)	0.92	1.07 (0.79–1.45)	0.65
Promised HK$200	1.09 (0.89–1.34)	0.46	1.23 (0.91–1.66)	0.18
Mixed incentive	1.14 (0.94–1.38)	0.20	1.33 (1.00–1.77)	0.05
Sex				
Female	Ref		–	–
Male	1.30 (1.08–1.57)	<0.01	–	–
Age group (years)		0.49		
15–29	Ref			
30–39	1.02 (0.75, 1.38)	0.90	–	–
40–49	1.02 (0.77, 1.36)	0.87	–	–
50–59	1.03 (0.78, 1.37)	0.84	–	–
60–65	0.78 (0.53, 1.15)	0.21	–	–
Above 65	1.05 (0.71, 1.55)	0.81	–	–
Education level		<0.01		0.08
Primary or below	Ref		Ref	
Secondary	1.45 (1.14–1.84)	<0.01	1.32 (0.98–1.79)	0.07
Tertiary	1.66 (1.27–2.17)	<0.01	1.47 (1.05–2.07)	0.03
Employment status		0.14		
Employed	Ref		–	–
Others	1.17 (0.93–1.49)	0.17	–	–
Retired	1.27 (0.95–1.68)	0.12	–	–
Had children under 16	1.28 (1.07–1.54)	<0.01	–	–
Place of birth		0.01		<0.01
Hong Kong	Ref		Ref	
China Mainland	0.80 (0.67–0.95)	<0.01	0.74 (0.58–0.93)	0.01
Other places	0.57 (0.32–1.00)	0.05	0.34 (0.12–1.01)	0.05
Intention to quit		<0.01		0.08
No intention	Ref		Ref	
Quit after 30 days	1.37 (1.13–1.65)	<0.01	1.13 (0.84–1.52)	0.43
Quit within 30 days	1.31 (1.11–1.54)	<0.01	1.30 (1.04–1.62)	0.02
Ever used Ecigs or HTP				
No	Ref		–	–
Yes	1.07 (0.89–1.28)	0.49	–	–

HK$100 = US$12.8; Ecigs or HTP: E-cigarettes or heated tobacco products; Baseline predictors shown in the table had *p*-value below 0.1 in the univariate analysis. Other variables with *p*-value 0.1 or higher were not included in the multivariate models

## Discussion

This RCT compared the effect of different incentive schemes on the consent and retention rates in a follow-up survey of adult cigarette smokers in Hong Kong, China. Our descriptive findings indicated that the consent and retention rates generally increased with the amount of incentive and were highest for the mixed incentive group. However, statistical significance was not achieved due to the small effect size and sample size. As no incentive was provided for the baseline survey, those who responded may tend to be altruistic rather than motivated by financial incentives. Such participants may therefore be less responsive to financial incentives for yet another survey. In comparison, in the International Tobacco Control Policy Evaluation Project [8, 33], an incentivized baseline survey, yielded higher retention rates for follow-up surveys. Some of our participants might also be overburdened by the baseline survey (about 15 min), which reduced their motivation to participate in a similar follow-up survey.

Our findings are consistent with those of trials that evaluated the effects of a prepaid incentive on survey participation [15, 30, 31, 34, 35]. Moreover, our results provided new evidence that the mixed incentive scheme increased the retention rate of baseline smokers for a follow-up survey compared with no incentive, although it was only marginally significant probably due to the small sample size. We have also provided empirical findings to support the aforementioned behavioral theories that rewards, particularly delivered early and unconditionally, can encourage participation or behavioral change [21, 36]. Respondents’ concerns about the credibility and confidentiality of the research may negatively affect trust. The unconditional and prepaid incentive as an alternative to establish trust may have decreased the respondents’ skepticism in our study, and this might have led to the greater 3-month retention.

The small effect size achieved by the mixed incentive scheme can be explained by the attrition rate in those who were told to receive the prepaid incentive. Only about half of the consented participants in the mixed incentive group were successfully contacted and given the prepaid $100 incentive. We showed that missing the chance of collecting the mailing address to deliver the incentive immediately after obtaining the consent would result in attrition in the subsequent follow-up and non-compliance of the intervention. Moreover, the retention rate in those who received the prepaid incentive in the mixed incentive group was significantly higher than that in the control group. This result indicates the importance of successfully and efficiently delivering the prepaid incentive for sustaining the intervention compliance. Apart from the traditional methods to mail the incentive, new payment methods that can increase the payment efficiency such as electronic transactions are recommended.

We found that higher education level and being a local resident (i.e., Hong Kong Chinese versus China Mainland Chinese and ethnic minority) were independently associated with greater consent and retention rates, which were consistent with previous findings [37–39]. Our results provide additional evidence that lower or no intention to quit led to higher attrition of a follow-up survey. Findings from follow-up surveys such as those on the change of smoking behaviors and attitudes may not be representative of all smoking respondents and may inflate the public support towards tobacco control policies [40–42]. Population-based cohort studies need to adjust the results by weighting for these factors appropriately.

The strength of our study was that the participants were recruited and the main baseline survey was started during the first “cold call” without prior invitation or mailed prepaid incentive. Therefore, we showed the incentive effect independent of other recruitment incentive and strategies. Our method also collected more personal information from those who did not consent to the follow-up in the first cold call, which allowed us to examine the factors associated with consent.

We found that higher education level and being a local resident (i.e., Hong Kong Chinese versus China Mainland Chinese and ethnic minority) were independently associated with greater consent and retention rates, which were consistent with previous findings [37–39]. Our results provide additional evidence that lower or no intention to quit led to higher attrition of a follow-up survey. Findings from follow-up surveys such as those on the change of smoking behaviors and attitudes may not be representative of all smoking respondents and may inflate the public support towards tobacco control policies [40–42]. Population-based cohort studies need to adjust the results by weighting for these factors appropriately.

Nonetheless, this trial had several limitations. First, the sample size was constrained by the limited recruitment period and preset sample size of the baseline survey. Second, this trial only enrolled smokers, and the findings may not be generalized to the overall population. Third, the use of postal service to send the incentive is a delayed mode of remuneration. Instant incentives have been shown to markedly increase the response rates than promised incentives among nonresponding physicians [43]. Given the rapidly evolving communication and technological landscape, future trials may consider using electronic payment methods to improve response rates.

## Conclusions

Our RCT showed that the proportion of smokers consenting to the 3-month follow-up and the retention rate generally increased with the incentive amount and offer of prepaid incentive. The combination of the prepaid and promised incentive (mixed incentive) was effective to increase the retention rate by 48%. We recommend this mixed incentive scheme to increase the follow-up retention rate. More efficient methods of delivering incentive are needed to maximize its effects.

## Data Availability

The data that support the findings of this study are available from Hong Kong Council on Smoking and Health, but restrictions apply to the availability of these data, which were used under license for the current study, and so are not publicly available. Data are however available from the authors upon reasonable request and with permission of Hong Kong Council on Smoking and Health.

## Abbreviations

ARR: Adjusted risk ratio
CI: Confidence interval
Ecigs: E-cigarettes
HTP: Heated tobacco products
RCT: Randomized controlled trial
RR: Risk ratio
SPSS: Statistical Package for the Social Sciences
TCPS: Tobacco Control Policy-related Survey
